# Clinical features modifying the cardiovascular benefits of GLP-1 receptor agonists: a systematic review and meta-analysis

**DOI:** 10.1093/ehjcvp/pvaf037

**Published:** 2025-08-31

**Authors:** Arzu Kalayci, James Louis Januzzi, Makiko Mitsunami, Ibrahim Halil Tanboga, Can Yucel Karabay, Charles Michael Gibson

**Affiliations:** Cardiovascular Division, Brigham and Women's Hospital, Harvard Medical School, 75 Francis Street, Boston, MA 02115, USA; Baim Institute for Clinical Research, 930 Commonwealth Avenue, Suite 3, Boston, MA 02215, USA; Baim Institute for Clinical Research, 930 Commonwealth Avenue, Suite 3, Boston, MA 02215, USA; Division of Cardiology, Department of Medicine, Massachusetts General Hospital, Harvard Medical School, 55 Fruit Street, Boston, MA 02114, USA; Department of Nutrition, Harvard T.H. Chan School of Public Health, 677 Huntington Avenue, Boston, MA 02115, USA; Department of Cardiology, Hisar Intercontinental Hospital, Nişantaşı University, Yamanevler, Site Yolu Cd No:7, 34768 Umraniye, Istanbul, Turkey; Department of Cardiology, Dr. Siyami Ersek Training and Research Hospital, University of Health Sciences, Selimiye, Tibbiye Cd No:25, 34668 Uskudar, Istanbul, Turkey; Baim Institute for Clinical Research, 930 Commonwealth Avenue, Suite 3, Boston, MA 02215, USA; Division of Cardiovascular Medicine, Department of Medicine, Beth Israel Deaconess Medical Center, Harvard Medical School, 330 Brookline Avenue, Boston, MA 02215, USA

**Keywords:** GLP-1 receptor agonists, Type 2 diabetes, Cardiovascular outcomes, Meta-analysis, Meta-regression, Effect modification

## Abstract

Glucagon-like peptide-1 receptor agonists (GLP-1 RAs) reduce major adverse cardiovascular events (MACE) in patients with type 2 diabetes (T2D), but heterogeneity exists across cardiovascular outcome trials (CVOTs). A comprehensive search of PubMed, EMBASE, and Cochrane Library was conducted through November 2024. Eligible CVOTs compared GLP-1 RAs with placebo in T2D patients. The primary outcome was MACE, defined as a composite of cardiovascular death, non-fatal myocardial infarction, and non-fatal stroke. Pooled odds ratios (ORs) with 95% confidence intervals (CIs) were calculated using a random-effects model. Heterogeneity was assessed using *I*², *τ*², and *R*². Meta-regression analyses evaluated the influence of baseline covariates on cardiovascular benefits of GLP-1 RAs, contingent upon the detection of moderate to substantial heterogeneity (*I*² ≥ 30%). Sensitivity analyses and GRADE assessments were also performed. Ten trials (67 769 patients; 34 536 receiving GLP-1 RAs) were analyzed. GLP-1 RAs significantly reduced MACE compared with placebo (OR = 0.87, 95% CI: 0.81–0.93, *P* < 0.001, *I*² = 48.4%). Cardiovascular death (OR = 0.86, 95% CI: 0.79–0.94, *P* < 0.001, *I*² = 22.6%) and all-cause mortality (OR = 0.87, 95% CI: 0.82–0.94, *P* < 0.001, *I*² = 17.7%) were also reduced. Meta-regression revealed a greater cardiovascular benefit in patients with higher baseline body mass index (BMI; logOR = −0.098 per kg/m², *P* = 0.006, *R*² = 99.98%) and older age (logOR = −0.033 per year, *P* = 0.023, *R*² = 75.47%). Sensitivity analyses confirmed the robustness of these findings, with consistent effect sizes and no single trial unduly influencing the results. The certainty of evidence was rated as high for all outcomes based on GRADE criteria. GLP-1 RAs significantly reduce MACE, cardiovascular death, and all-cause mortality in T2D patients. Higher baseline BMI and older age were associated with greater cardiovascular benefit.

## Graphical Abstract

**Graphical Abstract:**
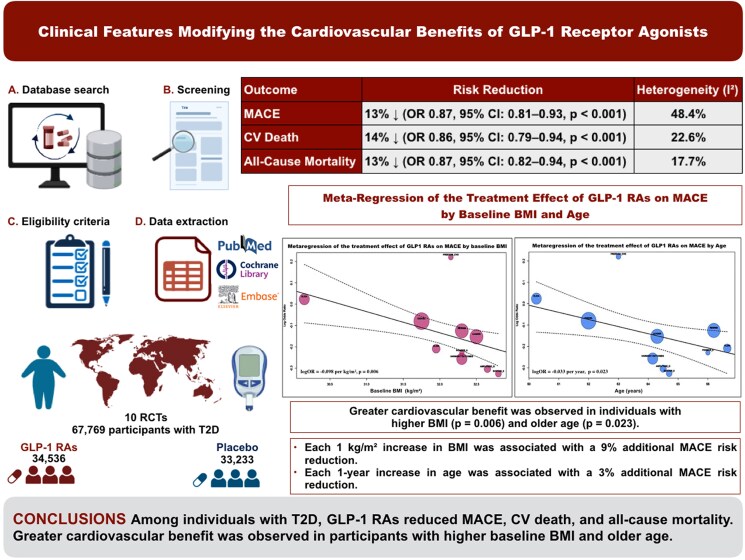

## Introduction

Type 2 diabetes (T2D) is a growing global health crisis, currently affecting over 530 million adults worldwide, with projections surpassing 640 million by 2030.^1,2^ Cardiovascular disease (CVD) remains the leading cause of death in patients with T2D, accounting for more than half of all diabetes-related mortality.^3,4^ The epidemic of obesity, affecting over 40% of adults in the United States,^5^ plays a central role in the pathogenesis of T2D and further amplifies cardiovascular risk. Obesity is not only a potent contributor to insulin resistance but also an independent risk factor for major adverse cardiovascular events (MACE), including myocardial infarction, stroke, and cardiovascular death.^6,7^ Together, T2D and obesity represent a complex metabolic cluster that significantly increases cardiovascular morbidity and mortality. While both conditions pose substantial challenges, obesity may be viewed as an upstream driver of the cardiometabolic burden observed in T2D populations.

In this context, glucagon-like peptide-1 receptor agonists (GLP-1 RAs) have emerged as a promising therapeutic class, offering dual benefits of glucose-lowering and cardiovascular risk reduction. Beyond their glucose-lowering effect, GLP-1 RAs have demonstrated weight loss, improved blood pressure, lipid profiles, and anti-inflammatory effects, suggesting pleiotropic mechanisms of cardiovascular protection. However, substantial variability in treatment effects has been observed across major GLP-1 RA cardiovascular outcome trials (CVOTs),^8–17^ raising concerns about the generalizability of findings.

In an effort to understand the heterogeneity observed in clinical trials of GLP-1 RAs, we conducted a meta-analysis of aggregate data from CVOTs and applied meta-regression methods to more comprehensively understand the cardiovascular benefits of GLP-1 RAs in patients with T2D. We hypothesized that heterogeneity of results from GLP-1 RA CVOTs might be explainable by identifiable differences in clinical makeup of the various trials.

## Methods

### Search strategy and data sources

This meta-analysis was conducted and reported in accordance with the Preferred Reporting Items for Systematic Reviews and Meta-Analyses (PRISMA) guidelines.^18^ This study was registered in the International Prospective Register of Systematic Reviews^19^ (PROSPERO) under the identification number CRD420251033082. Studies were eligible for inclusion if they were randomized controlled trials (RCTs) that included participants with T2D, compared a GLP-1 RA with placebo, and reported outcomes including MACE, cardiovascular mortality, and all-cause mortality.

A systematic search was conducted in MEDLINE (via PubMed), EMBASE, and the Cochrane Central Register of Controlled Trials (CENTRAL) from inception through November 2024. The search was limited to studies published in English and focused on identifying RCTs reporting cardiovascular outcomes in patients treated with GLP-1 RAs.

The detailed search strategy is provided in the *Supplementary file*, and the study selection process is illustrated in the PRISMA flowchart (Supplementary material online, *Figure S1*). The following terms were used to construct the search strategy: ‘glucagon-like peptide-1 receptor agonist’, ‘exenatide’, ‘liraglutide’, ‘lixisenatide’, ‘semaglutide’, ‘dulaglutide’, ‘albiglutide’, ‘efpeglenatide’, ‘placebo’, ‘MACE’, ‘cardiovascular mortality’, ‘myocardial infarction’, ‘all-cause mortality’, ‘stroke’, and ‘diabetes mellitus’. Boolean operators were applied to combine these terms systematically across all databases to ensure a comprehensive and thorough search. Duplicate records identified during the search were removed, and the remaining studies underwent title and abstract screening. Full texts of potentially eligible studies were reviewed based on predefined inclusion and exclusion criteria to ensure the final selection of relevant studies for the meta-analysis.

### Outcomes of interest

The primary outcome was MACE, defined as a composite of cardiovascular death, non-fatal myocardial infarction, and non-fatal stroke. Secondary outcomes included cardiovascular death and all-cause mortality.

### Data extraction and quality assessment

Two authors (A.K. and M.M.) independently screened the titles and abstracts of all identified studies, followed by a full-text review of shortlisted studies. Any disagreements regarding study eligibility were resolved through consultation with additional authors (I.H.T. and C.Y.K.). Data extraction was performed independently by two authors (A.K. and M.M.) using a standardized form. Extracted information included publication year, baseline participant characteristics, study drug, follow-up duration and the number of outcome events reported in each study. The risk of bias for each included study was assessed using the Cochrane Risk of Bias 2 (RoB 2) tool.^20^ The included trials were evaluated for potential bias across the domains of random sequence generation, allocation concealment, blinding, incomplete outcome data, and selective outcome reporting (see Supplementary material online, *Figure S2*).

### Statistical analysis

Pooled treatment effects were estimated using a random-effects model to account for variability across trials. Odds ratios (ORs) with 95% confidence intervals (CIs) were calculated for each outcome. Heterogeneity among studies was assessed using the *I*² statistic, *τ*², and Cochran’s *Q* test. Heterogeneity was interpreted based on *I*² values, with 25%, 50%, and 75% corresponding to low, moderate, and high heterogeneity, respectively.^21^ To further explore sources of heterogeneity, we performed meta-regression analyses to evaluate the moderating effects of baseline body mass index (BMI) and age on the treatment effects.

Meta-regression analyses were conducted using weighted least squares regression on the logarithm of the ORs, with baseline BMI and age as continuous covariates. The proportion of variability explained by these moderators (*R*²) was calculated. Statistical significance was defined as a *P*-value < 0.05. All analyses were conducted in R version 4.2.3 using the metafor package, a comprehensive tool for meta-analysis in R.

### GRADE assessment

We assessed the quality of evidence for each cardiovascular outcome using the GRADE^22^ (Grading of Recommendations, Assessment, Development and Evaluations) methodology. This framework classifies the certainty of evidence as high, moderate, low, or very low, based on a structured evaluation of study design, risk of bias, consistency of findings, directness of evidence, precision of effect estimates, and potential publication bias.

### Sensitivity analysis

To assess the robustness of the meta-analysis findings, we performed sensitivity analyses by sequentially excluding one study at a time and recalculating the pooled odds ratios for each outcome (leave-one-out approach). This method allowed us to determine whether the overall effect estimates were unduly influenced by any single trial. Stability of the results across these iterations was interpreted as evidence of robustness.

## Results

A total of 10 studies were included in the analysis, comprising 9 CVOTs and 1 kidney outcome trial (FLOW,^17^) as shown in *Table 1*. Although FLOW was primarily designed to evaluate kidney outcomes, it reported cardiovascular outcomes of interest and met the inclusion criteria. Together, these studies encompassed 67 769 persons with T2D, of whom 34 536 received GLP-1 RAs.

**Table 1:** Baseline characteristics of trials included in the meta-analysis of GLP-1 receptor agonists

Trial name	Year	Sample size (*n*)	GLP1 receptor agonist	Dose	Population	Median follow-up, months	Mean age, years	Male (%)	BMI, kg/m^2^	eGFR, mL/min/1.73 m^2^
ELIXA^8^	2015	6068	Lixisenatide	10 µg/day or 20 µg/day; subcutaneous	T2D and MI history	25	60.3	70	30.2 (5.7)	76 (21.4)
LEADER^9,23^	2016	9340	Liraglutide	1·8 mg/day; subcutaneous	T2D and high CV risk	45.6	64.3	64	32.5 (6.3)	80.4 (27.3)
SUSTAIN-6^10,24^	2016	3297	Semaglutide	0·5 mg/week or 1 mg/week; subcutaneous	T2D with or without previous CVD	25.2	64.6	61	32.8 (6.2)	76.1 (26.5)
EXSCEL^11^	2017	14 752	Exenatide	2 mg/week; subcutaneous	T2D with or without previous CVD	38.4	62	62	31.8 (5.9)	76.3 (22.8)
Harmony outcomes^12^	2018	9463	Albiglutide	30 mg/week or 50 mg/week; subcutaneous	T2D and CVD	19.2	64.2	70	32.3 (5.9)	79 (25.5)
REWIND^13^	2019	9901	Dulaglutide	1·5 mg//week; subcutaneous	T2D and previous CVD or high CV risk	64.8	66.2	54	32.3 (5.8)	74.9 (22)
PIONEER 6^14^	2019	3183	Semaglutide	14 mg/day; oral	T2D and high CV risk	15.9	66	68	32.3 (6.5)	74 (21)
AMPLITUDE-O^15^	2021	4076	Efpeglenatide	4 mg/week or 6 mg/week; subcutaneous	T2D with CVD or CKD and ≥ 1 CV risk factor	21.7	64.5	67	32.7 (6.2)	72.4 (22.4)
FREEDOM CVO^16^	2022	4156	Exenatide	20 µg/day for 3 months, then 60 µg/day; subcutaneous	T2D with CVD or CV risk	16	63	63	32.2	79.5 (18.5)
FLOW^17^	2024	3533	Semaglutide	1 mg/week; subcutaneous	T2D and CKD	40.8	66.6	70	32 (6.3)	47 (15.2)

Trial name	HbA1c (%)	LDL cholesterol, mg/dL	HDL cholesterol, mg/dL	Myocardial infarction, *n* (%)	Systolic blood pressure, mmHg	Heart failure history, *n* (%)	Atrial fibrillation, *n* (%)	Heart rate, beats/min
ELIXA^8^	7.7 (1.3)	78.5 (35.3)	43 (10.9)	1344 (22.1)	130 (17)	1358 (22.4)	366 (6)	70.2 (10)
LEADER^9,23^	8.7 (1.6)	NA	NA	2864 (30.7)	135.9 (17.8)	1667 (17.9)	NA	NA
SUSTAIN-6^10,24^	8.7 (1.5)	82.3 (45.6)	43.7 (27.1)	1072 (32.5)	135.6 (17.2)	777 (23.6)	NA	72 (10.9)
EXSCEL^11^	8.1 (1.9)	94.74	NA	NA	NA	2389 (16.2)	672 (4.6)	NA
Harmony Outcomes^12^	8.7 (1.5)	NA	NA	4459 (47)	134.8 (16.6)	1922 (20)	786 (8)	72
REWIND^13^	7.4 (1.1)	99 (37.9)	45.6 (13.5)	NA	137.2 (16.8)	853 (8.6)	NA	71.5 (10.9)
PIONEER 6^14^	8.2 (1.6)	78 (43.1)	40	1150 (36.1)	136 (18)	388 (12.2)	NA	NA
AMPLITUDE-O^15^	8.9 (1.5)	80.1 (37.9)	42.9 (12)	NA	134.9 (15.5)	737 (18.1)	NA	72.8 (10.6)
FREEDOM CVO^16^	8 (1.6)	NA	NA	1070 (25.7)	137.5 (14.4)	668 (16.1)	NA	NA
FLOW^17^	7.8 (1.3)	NA	NA	808 (22.9)	138.6 (15.8)	678 (19.2)	NA	NA

BMI, Body mass index; CKD, Chronic kidney disease; CV, Cardiovascular; CVD, Cardiovascular disease; eGFR, Estimated glomerular filtration rate; MI, Myocardial infarction; T2D, Type 2 diabetes.

HDL, high-density lipoprotein; LDL, low-density lipoprotein; NA, not available or not reported in the trial publication; values are presented as mean (SD).

**Fig. 1.**
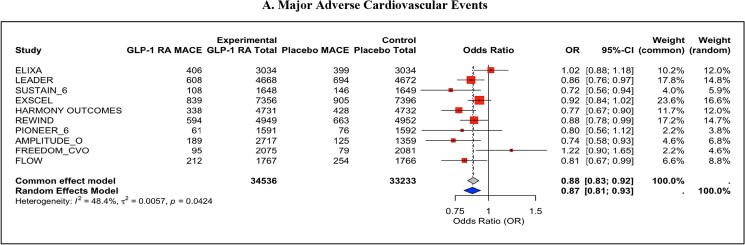
(*A*). Major adverse cardiovascular events.

**Fig. 2.**
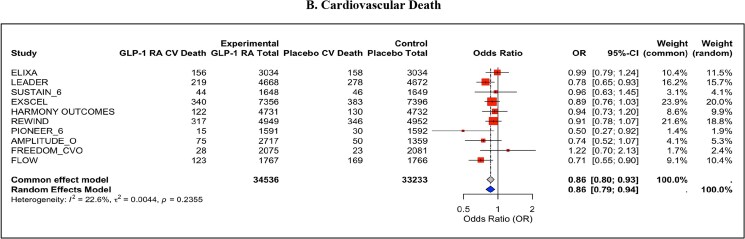
(*B*). Cardiovascular death.

**Fig. 3.**
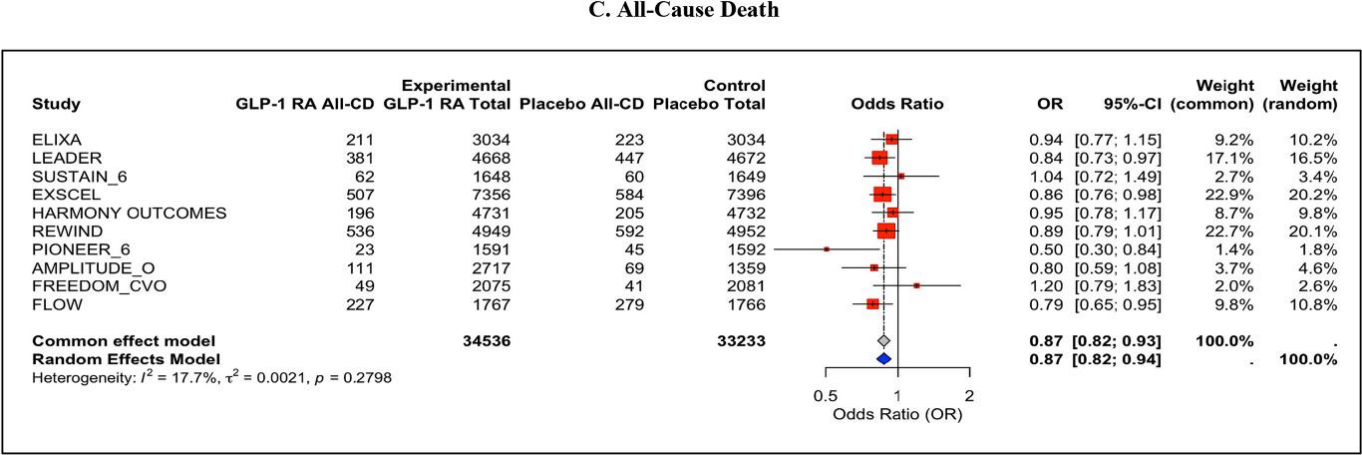
(*C*). All-cause death.

MACE was significantly reduced with GLP-1 RAs compared with placebo (OR = 0.87, 95% CI: 0.81–0.93, *P* < 0.001), with moderate heterogeneity observed (*I*² = 48.4%) (*Figure 1*). Secondary outcomes also demonstrated significant benefits; cardiovascular death was reduced by GLP-1 RAs (OR = 0.86, 95% CI: 0.79–0.94, *P* < 0.001) with low heterogeneity (*I*² = 22.6%) (*Figure 2*), and all-cause mortality was similarly reduced (OR = 0.87, 95% CI: 0.82–0.94, *P* < 0.001), with minimal heterogeneity (*I*² = 17.7%) (*Figure 3*).

**Fig. 4.**
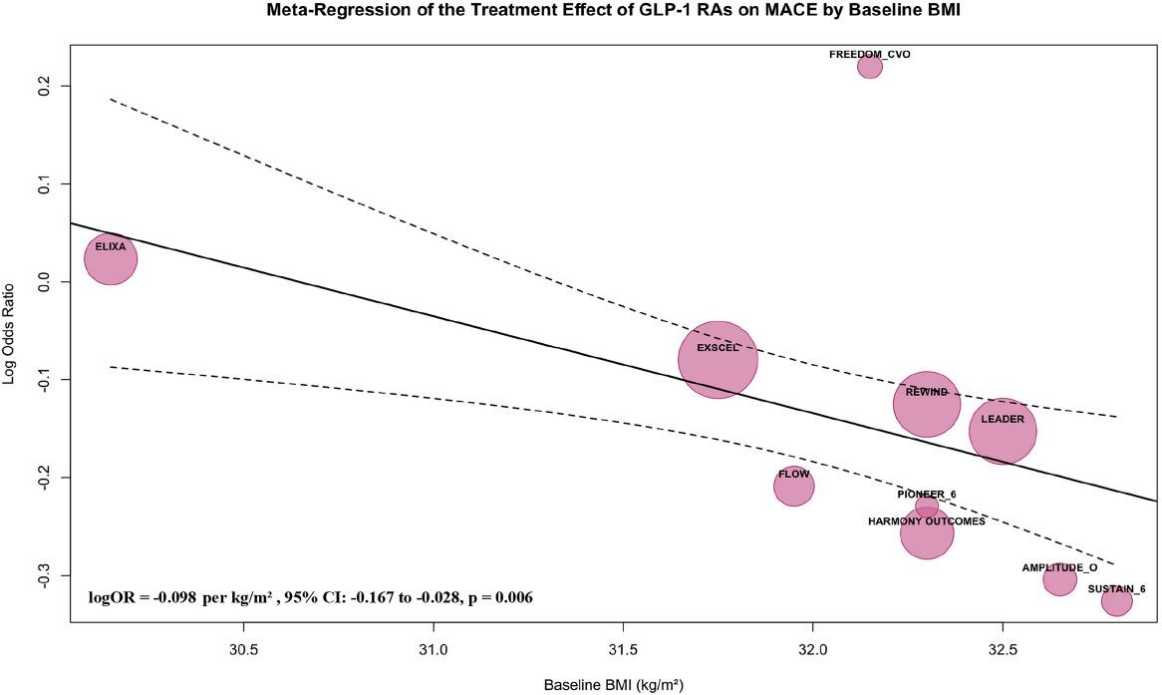
Meta-regression of the treatment effect of GLP-1 RAs on MACE by baseline BMI.

**Fig. 5.**
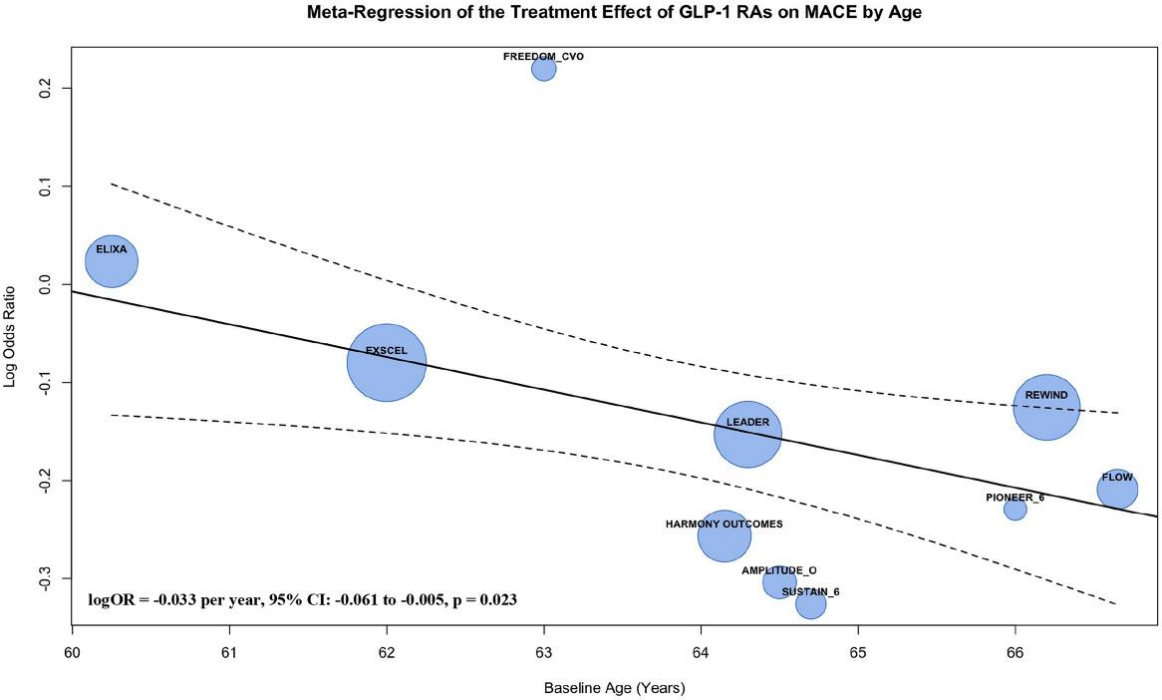
Meta-regression of the treatment effect of GLP-1 RAs on MACE by age.

To explore the sources of heterogeneity in MACE, meta-regression analyses were performed, revealing significant moderating effects of baseline BMI and age on the cardiovascular benefits of GLP-1 RAs. A significant inverse relationship between baseline BMI and cardiovascular benefit was observed (logOR = −0.098 per kg/m², 95% CI: −0.167 to −0.028, *P* = 0.006), indicating greater cardiovascular benefit in patients with higher baseline BMI. This effect explained nearly all of the observed heterogeneity (*R*² = 99.98%, QM = 7.61, *P* = 0.006). Similarly, baseline age moderated the treatment effect (logOR = −0.033 per year, 95% CI: −0.061 to −0.005, *P* = 0.023), with older patients deriving greater benefit from GLP-1 RAs. The influence of age accounted for 75.47% of the heterogeneity (*R*²), as supported by the meta-regression results (QM = 5.20, *P* = 0.023). Meta-regression bubble plots (*Figures 4 and 5*) further visualized the moderating effects of baseline BMI and age on the cardiovascular benefits of GLP-1 RAs.

To evaluate the robustness of the findings, sensitivity analyses were performed for each cardiovascular outcome by systematically excluding one trial at a time and recalculating the pooled odds ratios (leave-one-out method). For the primary outcome of MACE, the pooled odds ratio remained stable (OR range: 0.85–0.88), with heterogeneity (*I*²) estimates ranging from 38.0% to 54.1%, indicating moderate to substantial heterogeneity. For cardiovascular death and all-cause death, the direction and magnitude of effect remained consistent across all iterations (OR range: 0.87–0.89 and 0.84–0.88, respectively), with *I*² values ranging from 0% to 26.6% and 6.4% to 30.1%, respectively, reflecting low heterogeneity. These findings suggest that no single trial disproportionately influenced the overall results, supporting the reliability and robustness of the meta-analysis conclusions. The corresponding forest plots for these analyses are provided in the Supplementary material (Supplementary material online, *Figures S3*, *S4*, and *S5*).

Based on the GRADE approach, the certainty of evidence was rated as ‘High’ for all evaluated outcomes, including MACE, cardiovascular death, and all-cause death (*Table 2*). All included studies were RCTs with low risk of bias, and no serious inconsistency or imprecision was detected. Funnel plot inspection and Egger’s test (*P* > 0.10) revealed no indication of publication bias. Plausible confounding was unlikely, as all trials were placebo-controlled with adjudicated endpoints and similar baseline characteristics. Although effect sizes were directionally consistent and clinically relevant, they did not meet GRADE criteria for large or very large magnitude of effect. A formal dose-response analysis was not conducted, as most trials employed a single fixed dose. Overall, the evidence supporting the cardiovascular benefits of GLP-1 receptor agonists was considered robust, supported by a large sample size.

**Table 2:** GRADE evidence profile

Outcome	No. of studies	Study design	No. of participants	Risk of bias^a^	Inconsistency^b^	Imprecision^c^	Publication bias^d^	Plausible confounding^e^	Magnitude of effect^f^	Dose-response gradient^g^	Certainty^h^
MACE	10	RCT	67 769	No serious risk	No serious inconsistency	No serious imprecision	Undetected	Would not reduce effect	No	No	High
CV death	10	RCT	67 769	No serious risk	No serious inconsistency	No serious imprecision	Undetected	Would not reduce effect	No	No	High
All-cause death	10	RCT	67 769	No serious risk	No serious inconsistency	No serious imprecision	Undetected	would not reduce effect	No	No	High

^a^Risk of bias: Assessed using the Cochrane RoB 2 tool. All included trials were RCTs with low risk across key domains, including allocation concealment, blinding, and outcome assessment.

^b^Inconsistency: *I*² values were 48.4% for MACE, 22.6% for cardiovascular death, and 17.7% for all-cause mortality, indicating low to moderate heterogeneity that did not warrant downgrading.

^c^Imprecision: CIs were narrow and excluded the line of no effect for all outcomes. Sample sizes were large (*n* = 67 769), supporting the precision of effect estimates.

^d^Publication bias: Funnel plot inspection and Egger’s test (*P* > 0.10) revealed no significant evidence of small-study effects or publication bias.

^e^Plausible confounding: No major confounding expected; all included trials were placebo-controlled with similar baseline characteristics and adjudicated cardiovascular endpoints.

^f^Magnitude of effect: Although the direction and consistency of benefit were evident, effect sizes did not meet GRADE criteria for large or very large effects.

^g^Dose-response gradient: No dose-response relationship was observed across trials.

^h^Certainty rating: All outcomes were graded as high quality due to the robustness of RCT designs, consistency of findings, and large sample size.

## Discussion

This meta-analysis provides a comprehensive evaluation of the effects of GLP-1 RAs on cardiovascular outcomes in patients with T2D. The findings confirm that GLP-1 RAs significantly reduce the risk of MACE, CV death, and all-cause mortality compared with placebo. Specifically, our analysis revealed a 13% relative risk reduction in MACE, a 14% reduction in CV death, and a 13% reduction in all-cause mortality with GLP-1 RA use. Furthermore, extending the understanding of benefits from GLP-1 RA use in T2D, meta-regression analyses demonstrated that cardiovascular benefits were more pronounced in individuals with higher baseline BMI and older age, with incremental risk reductions in MACE. In particular, each 1 kg/m² increase in BMI was associated with an additional 9% MACE risk reduction, and each additional year of age was associated with a 3% MACE risk reduction. These findings underscore the importance of patient characteristics in explaining heterogeneity across GLP-1 RA trials.

Compared with previous meta-analyses,^25–27^ our study confirms the cardiovascular protective effects of GLP-1 RAs in individuals with T2D and further extends prior work by incorporating the most recent trials, increasing the overall sample size with a focus on T2D, and identifying important baseline characteristics—BMI and age—as significant modifiers of cardiovascular benefit.

Cardiovascular benefits of GLP-1 RAs are thought to result from a combination of mechanisms, including glycemic control, weight reduction, and broader metabolic effects such as improved endothelial function, reduced inflammation, and oxidative stress.^28–32^ Preclinical studies indicate that GLP-1 RAs contribute to cardiovascular protection by reducing atherosclerotic plaque burden, stabilising plaques, and mitigating myocardial ischemia-reperfusion injury.^29,33–35^ These mechanisms highlight their anti-inflammatory and cardiomyocyte-protective properties, which optimize cardiac energy metabolism and further mitigate adverse events like plaque rupture. Notably, prior findings suggest that baseline BMI does not consistently predict glycemic outcomes,^36^ indicating that the cardiovascular effects of GLP-1 RAs operate through distinct and multifaceted pathways beyond those influencing glucose regulation or weight loss. This complexity underscores their potential as targeted therapies addressing the diverse pathophysiology of CVD in T2D, particularly in high-risk populations.

The observed greater cardiovascular benefit of GLP-1 RAs in individuals with higher baseline BMI may be attributed to several mechanisms influenced by body weight. First, higher BMI may alter the pharmacokinetics and pharmacodynamics of GLP-1 RAs by affecting their distribution, metabolism, and elimination. Increased adiposity could enhance the distribution volume, potentially leading to prolonged drug exposure. Additionally, obesity-related changes in metabolic pathways^37^ and kidney function^38^ could modulate the breakdown and clearance of GLP-1 RAs. These factors may result in more sustained receptor activation and downstream signalling in individuals with higher BMI. Furthermore, higher BMI is often associated with a greater burden of metabolic and cardiovascular risk factors, which could make these patients more responsive to the pleiotropic effects of GLP-1 RAs, including weight reduction, anti-inflammatory effects, and improvements in endothelial function. These potential mechanisms suggest BMI may act as a moderating factor in the cardiovascular effects of GLP-1 RAs.

Our findings underscore the growing evidence supporting the role of GLP-1 RAs in reducing cardiovascular risk,^39^ particularly among individuals with greater metabolic and age-related vulnerabilities. The observed dose-response relationship between baseline BMI and age with cardiovascular efficacy aligns with recent trials showing that GLP-1 RAs may be especially effective in populations at elevated metabolic and cardiovascular risk such as those who have obesity and CVD.

To our knowledge, this is the first meta-analysis to identify baseline BMI and age as significant modifiers of the cardiovascular benefits of GLP-1 receptor agonists in individuals with T2Ds. These findings provide a nuanced understanding of patient subgroups who may derive the greatest benefit from therapy. In particular, patients with higher BMI appeared to gain incrementally greater cardiovascular protection, suggesting that beyond weight loss alone, GLP-1 RAs may offer enhanced cardiometabolic benefit in this population. This supports prioritising GLP-1 RAs in individuals with elevated BMI not just for weight reduction but also as a strategy to mitigate cardiovascular risk. Furthermore, the observed association between older age and greater treatment benefit challenges the perception of advanced age as a barrier to therapy. On the contrary, our results support broader use of GLP-1 RAs in older patients with T2D, a group often under-represented in clinical trials. Together, these findings open the door to future studies aimed at understanding the biological basis of effect modification and designing trials specifically powered to test GLP-1 RAs in specific subgroups. These findings may help guide clinical decision-making while also highlighting the need for precision-medicine approaches in future GLP-1 RA trials.

## Strengths and limitations

This analysis has several notable strengths. First, it includes large-scale RCTs that primarily report cardiovascular outcomes (with the exception of the FLOW trial,^17^) providing a robust evidence base. The large sample size enhances the statistical power and generalisability of the findings. Additionally, rigorous statistical methods were applied, including meta-regression, to investigate potential sources of heterogeneity and identify key moderating factors, supported by high certainty of evidence based on GRADE criteria. Sensitivity analyses using a leave-one-out approach further demonstrated the robustness of the results, with consistent effect estimates across all iterations, underscoring the stability of the findings. By focusing exclusively on trials conducted in individuals with T2D, the analysis maintains consistency across study populations, strengthening the internal validity of the conclusions.

Nonetheless, several limitations should be acknowledged. This meta-analysis relied on study-level (aggregate) data, which limited the ability to perform detailed subgroup analyses or adjust for individual-level covariates. There was inherent heterogeneity among the included GLP-1 RA trials, likely reflecting differences in trial design, populations, and endpoints. Notably, the definition of MACE varied slightly across trials: two studies (ELIXA^8^ and FREEDOM-CVO^16^) used a four-point MACE definition that included hospitalisation for unstable angina, while the remaining trials used the standard three-point definition. For consistency, we used the MACE event counts as reported in the original publications. Additionally, all included trials were sponsored by industry, which is a common feature of large-scale CVOTs. Importantly, the trial protocols and statistical analysis plans were publicly accessible, and outcomes were adjudicated by blinded assessors.

## Future directions

Future studies should aim to incorporate individual patient-level data to allow for more refined subgroup analyses and better adjustment for potential confounders. Such analyses could help confirm the observed modifying effects of BMI and age, and may also identify additional effect modifiers through more detailed investigation, including factors such as sex, ethnicity, renal function, and glycemic control. Moreover, further investigation into mechanistic pathways—particularly those independent of glycemic effects—is warranted to better understand how GLP-1 RAs confer cardiovascular benefits.

Finally, prospective trials specifically designed to evaluate GLP-1 RAs in high-risk subpopulations—such as older adults with obesity or heart failure—would help validate these findings, inform optimal dosing strategies, and support the development of personalized therapeutic approaches. These future efforts are especially important to avoid underutilisation of GLP-1 RAs in clinically vulnerable populations, where our findings suggest they may provide even greater cardiovascular benefit.

In conclusion, this meta-analysis demonstrates that GLP-1 RAs significantly reduce MACE, cardiovascular death, and all-cause mortality in individuals with T2D. Meta-regression findings suggest that these cardiovascular benefits are more pronounced in individuals with higher baseline BMI and older age. While these findings are based on study-level data, they underscore the relevance of patient-specific characteristics in shaping treatment response and highlight the need for future studies using individual-level data to confirm and expand upon these observations.

## Supplementary Material

pvaf037_Supplementary_Data

## Data Availability

This meta-analysis used only data extracted from publicly available RCTs. All data are available in the published articles cited in the manuscript.

## References

[1] International Diabetes Federation . IDF Diabetes Atlas. 11th ed. Brussels, Belgium: International Diabetes Federation; 2025.

[2] Centers for Disease Control and Prevention (CDC) . Type 2 Diabetes. Centers for Disease Control and Prevention. Published 2023. https://www.cdc.gov/diabetes/about/about-type-2-diabetes.html (20 April 2025).

[3] Rawshani A, Rawshani A, Franzén S, Sattar N, Eliasson B, Svensson AM, Zethelius B, Miftaraj M, McGuire DK, Rosengren A, Gudbjörnsdottir S. Risk factors, mortality, and cardiovascular outcomes in patients with type 2 diabetes. N Engl J Med 2018;379:633–644. 30110583 10.1056/NEJMoa1800256

[4] Raghavan S, Vassy JL, Ho YL, Song RJ, Gagnon DR, Cho K, Wilson PWF, Phillips LS. Diabetes mellitus-related all-cause and cardiovascular mortality in a national cohort of adults. J Am Heart Assoc 2019;8:e011295. 30776949 10.1161/JAHA.118.011295 PMC6405678

[5] Centers for Disease Control and Prevention (CDC) . Adult obesity prevalence remains high; support for prevention and treatment is critical. CDC Newsroom Published 2024. https://www.cdc.gov/media/releases/2024/p0912-adult-obesity.html (19 February 2025).

[6] Cercato C, Fonseca FA. Cardiovascular risk and obesity. Diabetol Metab Syndr 2019;11:74. 31467596 10.1186/s13098-019-0468-0 PMC6712750

[7] Dwivedi AK, Dubey P, Cistola DP, Reddy SY. Association between obesity and cardiovascular outcomes: updated evidence from meta-analysis studies. Curr Cardiol Rep 2020;22:25. 32166448 10.1007/s11886-020-1273-y PMC12285736

[8] Pfeffer MA, Claggett B, Diaz R, Dickstein K, Gerstein HC, Køber LV, Lawson FC, Ping L, Wei X, Lewis EF, Maggioni AP, McMurray JJ, Probstfield JL, Riddle MC, Solomon SD, Tardif JC; ELIXA Investigators. Lixisenatide in patients with type 2 diabetes and acute coronary syndrome. N Engl J Med 2015;373:2247–2257. 26630143 10.1056/NEJMoa1509225

[9] Marso SP, Daniels GH, Brown-Frandsen K, Kristensen P, Mann JF, Nauck MA, Nissen SE, Pocock S, Poulter NR, Ravn LS, Steinberg WM, Stockner M, Zinman B, Bergenstal RM, Buse JB; LEADER Steering Committee; LEADER Trial Investigators. Liraglutide and cardiovascular outcomes in type 2 diabetes. N Engl J Med 2016;375:311–322. 27295427 10.1056/NEJMoa1603827 PMC4985288

[10] Marso SP, Bain SC, Consoli A, Eliaschewitz FG, Jódar E, Leiter LA, Lingvay I, Rosenstock J, Seufert J, Warren ML, Woo V, Hansen O, Holst AG, Pettersson J, Vilsbøll T; SUSTAIN-6 Investigators. Semaglutide and cardiovascular outcomes in patients with type 2 diabetes. N Engl J Med 2016;375:1834–1844. 27633186 10.1056/NEJMoa1607141

[11] Holman RR, Bethel MA, Mentz RJ, Thompson VP, Lokhnygina Y, Buse JB, Chan JC, Choi J, Gustavson SM, Iqbal N, Maggioni AP, Marso SP, Öhman P, Pagidipati NJ, Poulter N, Ramachandran A, Zinman B, Hernandez AF EXSCEL Study Group. Effects of once-weekly exenatide on cardiovascular outcomes in type 2 diabetes. N Engl J Med 2017;377:1228–1239. 28910237 10.1056/NEJMoa1612917 PMC9792409

[12] Green JB, Hernandez AF, D'Agostino RB, Granger CB, Janmohamed S, Jones NP, Leiter LA, Noronha D, Russell R, Sigmon K, Del Prato S, McMurray JJV. Harmony outcomes: a randomized, double-blind, placebo-controlled trial of the effect of albiglutide on major cardiovascular events in patients with type 2 diabetes mellitus. Am Heart J 2018;203:30–38. 30015066 10.1016/j.ahj.2018.03.030

[13] Gerstein HC, Colhoun HM, Dagenais GR, Diaz R, Lakshmanan M, Pais P, Probstfield J, Riesmeyer JS, Riddle MC, Rydén L, Xavier D, Atisso CM, Dyal L, Hall S, Rao-Melacini P, Wong G, Avezum A, Basile J, Chung N, Conget I, Cushman WC, Franek E, Hancu N, Hanefeld M, Holt S, Jansky P, Keltai M, Lanas F, Leiter LA, Lopez-Jaramillo P, Cardona Munoz EG, Pirags V, Pogosova N, Raubenheimer PJ, Shaw JE, Sheu WH, Temelkova-Kurktschiev T; REWIND Investigators. Dulaglutide and cardiovascular outcomes in type 2 diabetes (REWIND): a double-blind, randomised placebo-controlled trial. Lancet 2019;394:121–130. 31189511 10.1016/S0140-6736(19)31149-3

[14] Husain M, Birkenfeld AL, Donsmark M, Dungan K, Eliaschewitz FG, Franco DR, Jeppesen OK, Lingvay I, Mosenzon O, Pedersen SD, Tack CJ, Thomsen M, Vilsbøll T, Warren ML, Bain SC; PIONEER 6 Investigators. Oral semaglutide and cardiovascular outcomes in patients with type 2 diabetes. N Engl J Med 2019;381:841–851. 31185157 10.1056/NEJMoa1901118

[15] Gerstein HC, Sattar N, Rosenstock J, Ramasundarahettige C, Pratley R, Lopes RD, Lam CSP, Khurmi NS, Heenan L, Del Prato S, Dyal L, Branch K; AMPLITUDE-O Trial Investigators. Cardiovascular and renal outcomes with efpeglenatide in type 2 diabetes. N Engl J Med 2021;385:896–907. 34215025 10.1056/NEJMoa2108269

[16] Ruff CT, Baron M, Im K, O'Donoghue ML, Fiedorek FT, Sabatine MS. Subcutaneous infusion of exenatide and cardiovascular outcomes in type 2 diabetes: a non-inferiority randomized controlled trial. Nat Med 2022;28:89–95. 34873344 10.1038/s41591-021-01584-3

[17] Perkovic V, Tuttle KR, Rossing P, Mahaffey KW, Mann JFE, Bakris G, Baeres FMM, Idorn T, Bosch-Traberg H, Lausvig NL, Pratley R; FLOW Trial Committees and Investigators. Effects of semaglutide on chronic kidney disease in patients with type 2 diabetes. N Engl J Med 2024;391:109–121. 38785209 10.1056/NEJMoa2403347

[18] Page MJ, McKenzie JE, Bossuyt PM, Boutron I, Hoffmann TC, Mulrow CD, Shamseer L, Tetzlaff JM, Akl EA, Brennan SE, Chou R, Glanville J, Grimshaw JM, Hróbjartsson A, Lalu MM, Li T, Loder EW, Mayo-Wilson E, McDonald S, McGuinness LA, Stewart LA, Thomas J, Tricco AC, Welch VA, Whiting P, Moher D. The PRISMA 2020 statement: an updated guideline for reporting systematic reviews. BMJ 2021;372:n71. 33782057 10.1136/bmj.n71 PMC8005924

[19] Sideri S, Papageorgiou SN, Eliades T. Registration in the international prospective register of systematic reviews (PROSPERO) of systematic review protocols was associated with increased review quality. J Clin Epidemiol 2018;100:103–110. 29339215 10.1016/j.jclinepi.2018.01.003

[20] Higgins JP, Altman DG, Gøtzsche PC, Jüni P, Moher D, Oxman AD, Savovic J, Schulz KF, Weeks L, Sterne JA; Cochrane Bias Methods Group; Cochrane Statistical Methods Group. The Cochrane Collaboration's tool for assessing risk of bias in randomized trials. BMJ 2011;343:d5928. 22008217 10.1136/bmj.d5928 PMC3196245

[21] Higgins JP, Thompson SG, Deeks JJ, Altman DG. Measuring inconsistency in meta-analyses. BMJ 2003;327:557–560. 12958120 10.1136/bmj.327.7414.557 PMC192859

[22] Guyatt GH, Oxman AD, Vist GE, Kunz R, Falck-Ytter Y, Alonso-Coello P, Schünemann HJ; GRADE Working Group. GRADE: an emerging consensus on rating quality of evidence and strength of recommendations. BMJ 2008;336:924–926. 18436948 10.1136/bmj.39489.470347.AD PMC2335261

[23] Mann JFE, Ørsted DD, Brown-Frandsen K, Marso SP, Poulter NR, Rasmussen S, Tornøe K, Zinman B, Buse JB; LEADER Steering Committee and Investigators. Liraglutide and renal outcomes in type 2 diabetes. N Engl J Med 2017;377:839–848. 28854085 10.1056/NEJMoa1616011

[24] Mann JFE, Hansen T, Idorn T, Leiter LA, Marso SP, Rossing P, Seufert J, Tadayon S, Vilsbøll T. Effects of once-weekly subcutaneous semaglutide on kidney function and safety in patients with type 2 diabetes: a post-hoc analysis of the SUSTAIN 1-7 randomised controlled trials. Lancet Diabetes Endocrinol 2020;8:880–893. 32971040 10.1016/S2213-8587(20)30313-2

[25] Chen X, Zhang X, Xiang X, Fang X, Feng S. Effects of glucagon-like peptide-1 receptor agonists on cardiovascular outcomes in high-risk type 2 diabetes: a systematic review and meta-analysis of randomized controlled trials. Diabetol Metab Syndr 2024;16:251. 39456002 10.1186/s13098-024-01497-4 PMC11515276

[26] Badve SV, Bilal A, Lee MMY, Sattar N, Gerstein HC, Ruff CT, McMurray JJV, Rossing P, Bakris G, Mahaffey KW, Mann JFE, Colhoun HM, Tuttle KR, Pratley RE, Perkovic V. Effects of GLP-1 receptor agonists on kidney and cardiovascular disease outcomes: a meta-analysis of randomised controlled trials. Lancet Diabetes Endocrinol 2025;13:15–28. 39608381 10.1016/S2213-8587(24)00271-7

[27] Rivera FB, Cruz LLA, Magalong JV, Ruyeras JMMJ, Aparece JP, Bantayan NRB, Lara-Breitinger K, Gulati M. Cardiovascular and renal outcomes of glucagon-like peptide 1 receptor agonists among patients with and without type 2 diabetes mellitus: a meta-analysis of randomized placebo-controlled trials. Am J Prev Cardiol 2024;18:100679. 38779187 10.1016/j.ajpc.2024.100679 PMC11108827

[28] Sharma A, Verma S. Mechanisms by which glucagon-like peptide-1 receptor agonists and sodium-glucose cotransporter-2 inhibitors reduce cardiovascular risk in adults with type 2 diabetes mellitus. Can J Diabetes 2020;44:93–102. 31882322 10.1016/j.jcjd.2019.09.003

[29] Gaspari T, Welungoda I, Widdop RE, Simpson RW, Dear AE. The GLP-1 receptor agonist liraglutide inhibits progression of vascular disease via effects on atherogenesis, plaque stability and endothelial function in an ApoE (-/-) mouse model. Diab Vasc Dis Res 2013;10:353–360. 23673376 10.1177/1479164113481817

[30] Rakipovski G, Rolin B, Nøhr J, Klewe I, Frederiksen KS, Augustin R, Hecksher-Sørensen J, Ingvorsen C, Polex-Wolf J, Knudsen LB. The GLP-1 analogs liraglutide and semaglutide reduce atherosclerosis in ApoE−/− and LDLr−/− mice by a mechanism that includes inflammatory pathways. JACC Basic Transl Sci 2018;3:844–857. 30623143 10.1016/j.jacbts.2018.09.004 PMC6314963

[31] Liu H, Dear AE, Knudsen LB, Simpson RW. A long-acting glucagon-like peptide-1 analogue attenuates induction of plasminogen activator inhibitor type-1 and vascular adhesion molecules. J Endocrinol 2009;201:59–66. 19136619 10.1677/JOE-08-0468

[32] Dokken BB, Hilwig WR, Teachey MK, Panchal RA, Hubner K, Allen D, Rogers DC, Kern KB. Glucagon-like peptide-1 (GLP-1) attenuates post-resuscitation myocardial microcirculatory dysfunction. Resuscitation 2010;81:755–760. 20347207 10.1016/j.resuscitation.2010.01.031

[33] Noyan-Ashraf MH, Momen MA, Ban K, Sadi AM, Zhou YQ, Riazi AM, Baggio LL, Henkelman RM, Husain M, Drucker DJ. GLP-1R agonist liraglutide activates cytoprotective pathways and improves outcomes after experimental myocardial infarction in mice. Diabetes 2009;58:975–983. 19151200 10.2337/db08-1193 PMC2661586

[34] Sonne DP, Engstrøm T, Treiman M. Protective effects of GLP-1 analogues exendin-4 and GLP-1(9–36) amide against ischemia-reperfusion injury in rat heart. Regul Pept 2008;146:243–249. 17976835 10.1016/j.regpep.2007.10.001

[35] Bose AK, Mocanu MM, Carr RD, Brand CL, Yellon DM. Glucagon-like peptide 1 can directly protect the heart against ischemia/reperfusion injury. Diabetes 2005;54:146–151. 15616022 10.2337/diabetes.54.1.146

[36] Cai X, Yang W, Gao X, Zhou L, Han X, Ji L. Baseline body mass index and the efficacy of hypoglycemic treatment in type 2 diabetes: a meta-analysis. PLoS One 2016;11:e0166625. 27935975 10.1371/journal.pone.0166625 PMC5147850

[37] Sam S, Mazzone T. Adipose tissue changes in obesity and the impact on metabolic function. Transl Res 2014;164:284–292. 24929206 10.1016/j.trsl.2014.05.008

[38] Tsuboi N, Okabayashi Y, Shimizu A, Yokoo T. The renal pathology of obesity. Kidney Int Rep 2017;2:251–260. 29142961 10.1016/j.ekir.2017.01.007 PMC5678647

[39] Schütt K, Federici M, Verket M, Marx N, Müller-Wieland D. 2023 ESC guidelines for the management of cardiovascular disease in patients with diabetes. Eur Heart J 2023;44:4043–4140. 38195096 10.1093/eurheartj/ehad881

