# Supplementary material for: Clinical features modifying the cardiovascular benefits of GLP-1 receptor agonists: a systematic review and meta-analysis
Source: Eur Heart J Cardiovasc Pharmacother. 2025 Aug 31;11(6):552–61. doi: 10.1093/ehjcvp/pvaf037 (PMC12450594; doi:10.1093/ehjcvp/pvaf037)
Supplement: pvaf037_Supplementary_Data [file pvaf037_supplementary_data.docx]

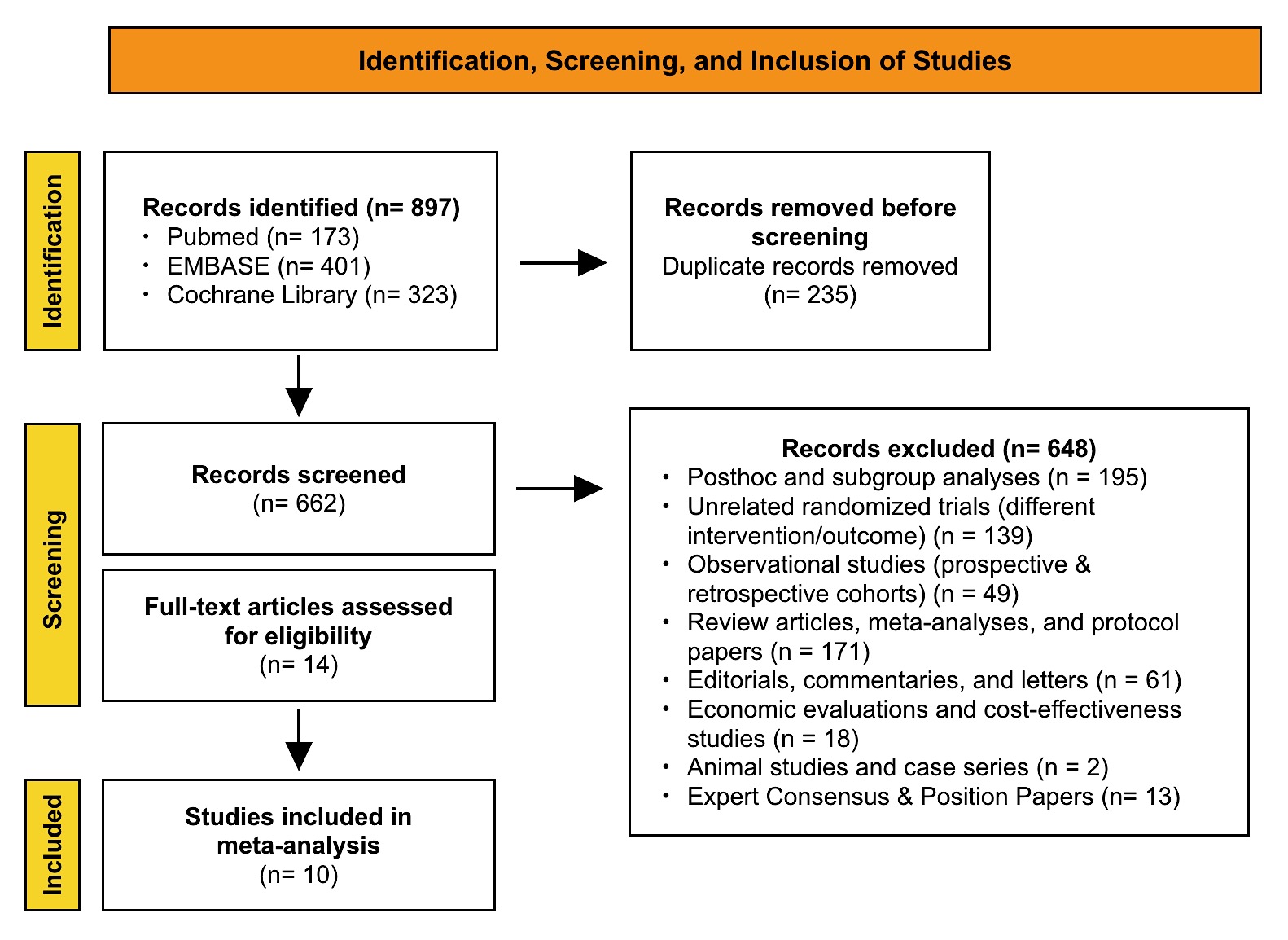
**Supplementary Figure 1:** PRISMA Flow Diagram of Study Selection


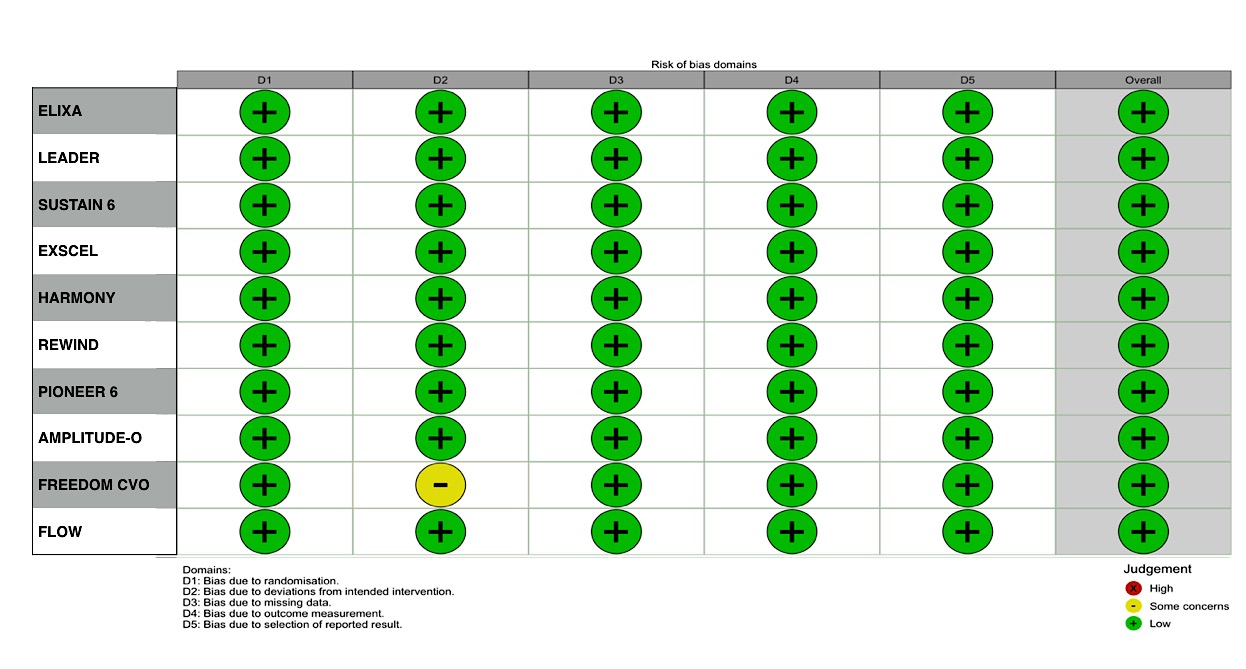
**Supplementary Figure 2:** Risk of bias assessment


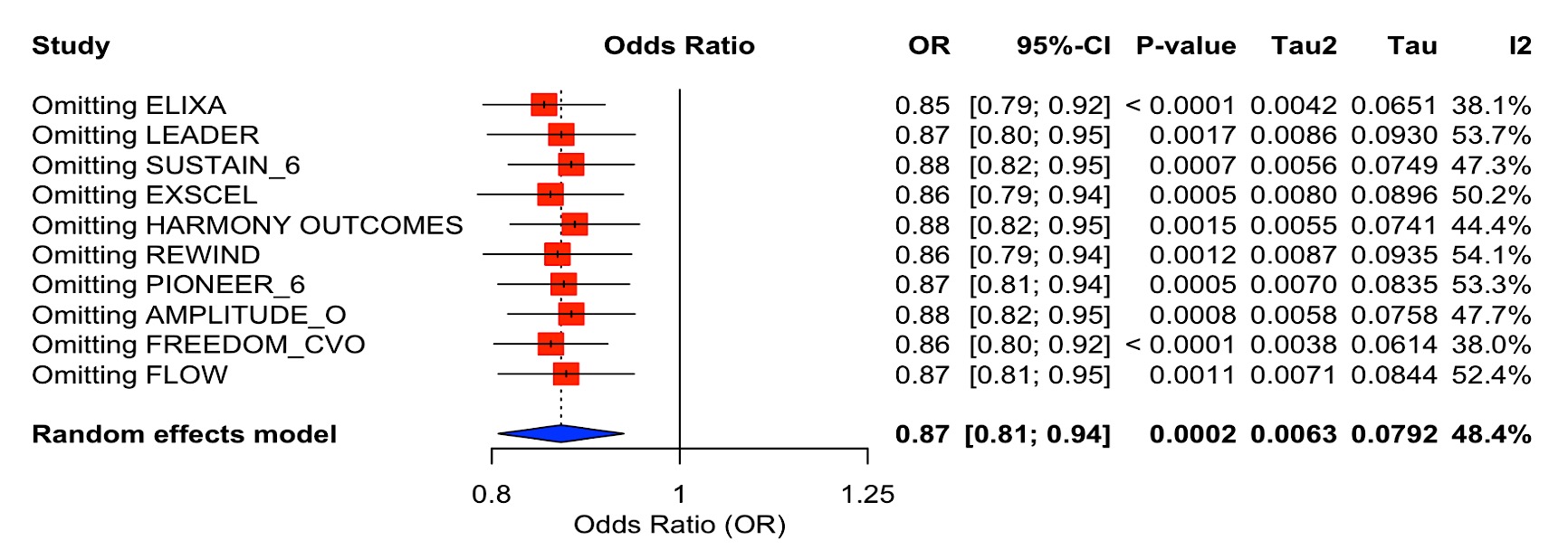
**Supplementary Figure 3.** Leave-one-out sensitivity analysis for MACE


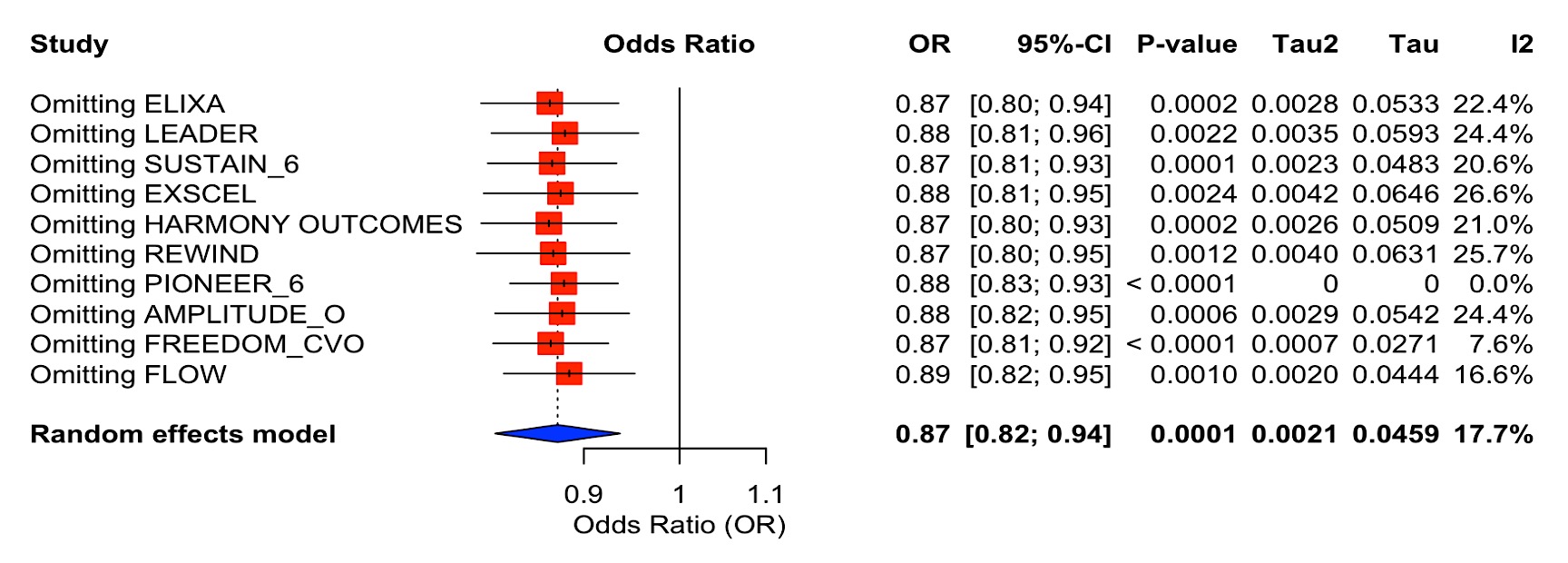
**Supplementary Figure 4.** Leave-one-out sensitivity analysis for cardiovascular death


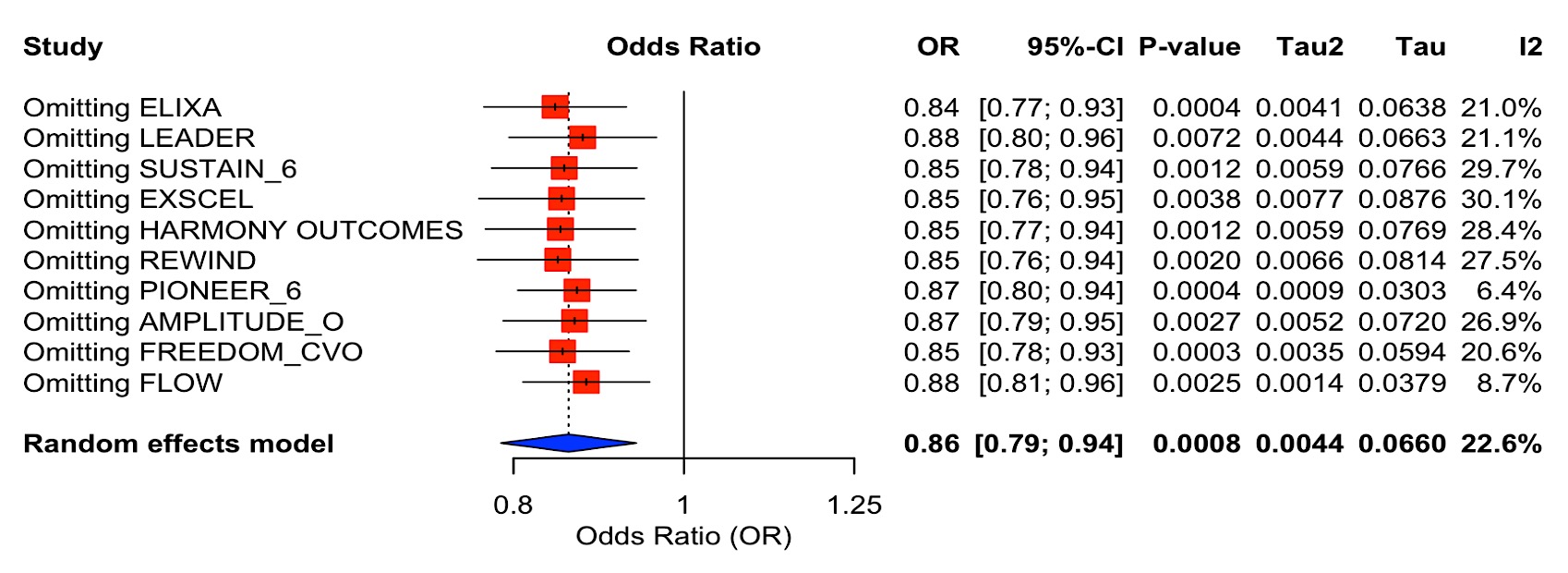
**Supplementary Figure 5.** Leave-one-out sensitivity analysis for all-cause death
